# Exploring the Congruence Between Age Friendly Practice and Perceptions in a College of Nursing

**DOI:** 10.1093/geroni/igaf122.2144

**Published:** 2025-12-31

**Authors:** Jacqueline Eaton, Katarina Friberg-Felsted, Rebekah Perkins, Joshua Seabury, Kara Dassel, Sara Hart

**Affiliations:** University of Utah, Salt Lake City, Utah, United States; University of Utah, Salt Lake City, Utah, United States; University of Utah, Salt Lake City, Utah, United States; University of Utah, Salt Lake City, Utah, United States; University of Utah, Salt Lake City, Utah, United States; University of Utah, Salt Lake City, Utah, United States

## Abstract

Fostering an age-friendly environment is imperative to gerontology and nursing education. In an effort to advance University of Utah College of Nursing (CON) educational excellence, we implemented the Age-Friendly Inventory (AFI) and Campus Climate Survey (ICCS) to identify strengths and weaknesses in our College. The purpose of this presentation is to describe age friendly practices across the University of Utah and how these align with perceptions from CON students, faculty, and staff. Fourteen units across campus completed individual sections of the AFI. CCS surveys were distributed electronically to gather CON perceptions of age-friendly practices (N = 240). The AFI was analyzed to identify practices across seven domains. Congruence between AFI and CCS responses was analyzed to identify agreement between awareness and practice. Age friendly practices were most present in the domain of Physical Environment (94%), and Personnel (78%) and least present in Teaching and Learning (50%). Overall congruence demonstrated low match between practice and perception (M = 0.37, SD = 0.13). The highest congruence was faculty response to the Research domain (M = 0.70, SD = 0.40) while the lowest congruence was staff response to the domain of Outreach and Engagement (M = 0.06, SD = 0.09). Practice and perception alignment was low overall, demonstrating a need to improve awareness of age-friendly practices across faculty, staff, and students in the CON. We are using these results to create professional development opportunities targeting CON needs.

